# Transcriptome-wide analysis reveals different categories of response to a standardised immune challenge in a wild rodent

**DOI:** 10.1038/s41598-020-64307-7

**Published:** 2020-05-04

**Authors:** Klara M. Wanelik, Mike Begon, Elena Arriero, Janette E. Bradley, Ida M. Friberg, Joseph A. Jackson, Christopher H. Taylor, Steve Paterson

**Affiliations:** 1 Institute of Integrative Biology, University of Liverpool, Liverpool, United Kingdom; 2 School of Life Sciences, University of Nottingham, Nottingham, United Kingdom; 3 Department of Biodiversity, Ecology and Evolution, University Complutense of Madrid, Madrid, Spain; 4 School of Environment and Life Sciences, University of Salford, Salford, United Kingdom

**Keywords:** Molecular ecology, Gene expression, Immunogenetics, Prognostic markers

## Abstract

Individuals vary in their immune response and, as a result, some are more susceptible to infectious disease than others. Little is known about the nature of this individual variation in natural populations, or which components of immune pathways are most responsible, but defining this underlying landscape of variation is an essential first step to understanding the drivers of this variation and, ultimately, predicting the outcome of infection. We describe transcriptome-wide variation in response to a standardised immune challenge in wild field voles. We find that genes (hereafter 'markers') can be categorised into a limited number of types. For the majority of markers, the response of an individual is dependent on its baseline expression level, with significant enrichment in this category for conventional immune pathways. Another, moderately sized, category contains markers for which the responses of different individuals are also variable but independent of their baseline expression levels. This category lacks any enrichment for conventional immune pathways. We further identify markers which display particularly high individual variability in response, and could be used as markers of immune response in larger studies. Our work shows how a standardised challenge performed on a natural population can reveal the patterns of natural variation in immune response.

## Introduction

Individuals vary in their immune response. Within a population, some individuals may fail to make protective immune responses following either natural infection or vaccination and so are especially vulnerable to infectious disease^1–4^. Defining the patterns of such variability will enhance our ability to manage the health of individuals – especially those that are most susceptible to infectious disease in human, livestock or wildlife populations.

Studies in laboratory mice are the cornerstone of immunology and have provided a detailed understanding of the molecular and cellular pathways by which immune responses are effected. This impressive mechanistic understanding, however, has only been achieved by minimising genetic and environmental variation within a laboratory setting. Where laboratory studies have examined the effects of variability – in genetics, microbiota or diet – both qualitative and quantitative differences in immune responses have been observed, with consequent effects on infection^5–7^. Nevertheless, natural variability cannot be fully reproduced in the laboratory, which has led to a recent effort to characterise the immune response in wild populations of mice or other rodents. Recent work in mice from agricultural and other anthropogenic settings is consistent with the expectation that exposure to complex environments greatly alters immune function^8^. New populations of memory T cells, present only in non-laboratory mice, have also been identified^9^.

One commonly used measure of an immune response is to assess the amount of one or more markers (e.g. transcripts or proteins) produced by a population of cells following stimulation by an immune agonist. From this *ex vivo* assay, one can gain insight into the types of immune response that could be made to a pathogen *in vivo*. Such responses will of course depend on the cell type, the time point and the immune agonist used. Nevertheless, for any molecular marker, individuals, in natural populations especially, could exhibit different marker abundances prior to and/or following stimulation (hereafter ‘baseline’ and ‘stimulated’ abundances respectively), leading to differences in their response to stimulation, and likely differences in their ability to control infection. An individual’s response to stimulation is here defined as the difference between its baseline and stimulated abundance of a particular marker. Furthermore, we argue that the most useful (and interesting) markers, in terms of understanding why individuals vary in their ability to mount a successful immune response, are likely to be those for which response to stimulation is most variable among individuals.

We use a wild population of field voles (*Microtus agrestis*) to examine naturally occurring patterns of individual variation in response to stimulation, across the transcriptome, as an essential first step towards understanding the processes driving these patterns. Given that we study a mixed population of cells (splenocytes), variation in the responses we measure could result from both the relative proportion and activation state of different cell types in different individual animals. Each of these things could, potentially, be driven independently by different causal drivers in individual animals and, furthermore, both could affect the strength and phenotypic direction of any response. Nonetheless, such composite responses are still likely to map onto functional responses and to represent a useful marker of individual variability.

**Figure 1:**
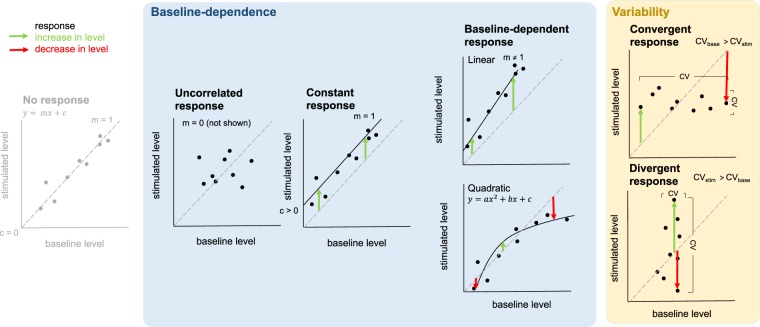
Different categories of response to a standardised immune challenge. These are based on two overlapping sets of criteria: baseline-dependence of an individual’s response (blue background) and individual variability in response (yellow background). Arrows represent individual responses. No response (for reference): markers for which individuals (on average) show no response to stimulation (intercept not significantly different from zero; slope not significantly different from one). Uncorrelated response: markers for which responses of different individuals are variable and independent of their baseline (slope not significantly different from zero). Constant response: markers for which the responses of different individuals are (on average) constant and independent of their baseline (intercept significantly greater than zero; slope not significantly different from one). Baseline-dependent response: markers for which responses of different individuals vary as a function of their baseline, either as a linear function of their baseline (slope significantly different from one; slope greater than one is depicted but could equally be less than one), or as a quadratic function of their baseline (a saturating function is depicted but could equally be exponential). Convergent response: markers for which the coefficient of variation (CV) for baseline abundances is significantly greater than the CV for stimulated abundances across individuals (CV_base_> CV_stim_). Divergent response: markers for which the CV for stimulated abundances is significantly greater than the CV for baseline abundances across individuals (CV_stim_> CV_base_). Both convergent and divergent markers depicted as, but not limited to, markers for which response is uncorrelated.

We describe three main categories of response to stimulation: (i) uncorrelated response, (ii) constant response and (iii) baseline-dependent response (depicted in Fig. 1). We also identify markers, across these three categories, which show particularly high individual variability in response. We suggest that such categorisation is useful in organising natural variation in response, since little is known about which components of immune pathways are responsible for natural variability in immune response, or about the nature of such variability. Indeed, this categorisation is not limited to the components of conventional immune pathways. The ability of an immune response to effect protection against infection, for example, will be supported by a variety of non-immune functions, that will also be activated following stimulation by an agonist, and vary to a greater or lesser extent among individuals within a natural population. By identifying the components (whether conventionally immunological or not) that are likely to be responsible for natural variability in immune response, and by describing the nature of their variability, we are laying the groundwork for exploring the processes, whether genetic or environmental, which drive individual variation in immune response.

## Methods

### Field methods

Sixty-two field voles were collected between July and October 2015 to assay expression by RNASeq. Voles came from four sites in Kielder Forest, Northumberland (55° 13′ N, 2° 33′ W). Each site contained a trapping grid of regularly spaced traps (at approximately 5 m intervals) and was also used for other components of a larger field study (for more details see^10^).

#### Ethics statement

All animal procedures carried out as part of this larger field study were performed with approval from the University of Liverpool Welfare Committee and the UK Home Office (PPL 70/8210), and in accordance with the Animals (Scientific Procedures) Act 1986.

### Laboratory methods

Following collection, voles were transported to the laboratory where they were weighed, aged (as either mature or immature) and sexed. Maturity (or immaturity) was determined ultimately following dissection. Males with scrotal testes and females with perforate vaginas were classified as mature. Non-scrotal males and non-perforate females can either be immature, or mature but sexually inactive. In these cases a consensus classification based on the internal reproductive organs, coat colour and size was used. Sampled voles varied in body weight (Median = 22.5 g; 25% centile = 18.8 g; 75% centile = 30.2 g) and included both mature (*n* = 43) and immature (*n* = 19) individuals. As our UK Home Office licence did not allow us to collect overtly pregnant females, there are fewer females (*n* = 18) than males (*n* = 44) in our sample. Voles were killed by a rising concentration of CO_2_ followed by exsanguination.

#### Splenocyte cultures

The whole spleen was removed from each vole, and a splenocyte culture was established from this. The splenocyte culture from each vole was split into two populations, one of which was stimulated with anti-CD3 antibodies (Hamster Anti-Mouse CD3e, Clone 500A2 from BD Pharmingen) and anti-CD28 antibodies (Hamster Anti-Mouse CD28, Clone 37.51 from BD Tombo Biosciences) at concentrations of 2 μg/ml and of 1 μg/ml respectively for 24 hr, and the other was left as an unstimulated control to act as a reference level. We refer to this reference level as the 'baseline', and control samples as 'baseline' samples. However, it is important to note that this level will vary for an individual, not only on a day to day basis, but throughout its life. Cell culture conditions were otherwise equivalent to those used in Jackson *et al.*^11^. Costimulation with anti-CD3 and anti-CD28 antibodies was used to selectively promote the proliferation of T cells^12^. We assumed that this would reflect the potential response of T-cell populations, a central process contributing to complex immune responses against invading pathogens, *in vivo*. We did not count the numbers of cells of different types (e.g. T cells, B cells or monocytes) in each spleen, but expect this to vary between spleens and therefore between splenocyte cultures.

#### RNASeq preparation and mapping

RNA was extracted from splenocyte cultures using Invitrogen PureLink kits. The amount of total RNA, and A_260_/A_280_ and A_260_/A_230_ nm ratios were assessed using the NanoDrop 1000 (Thermo Fisher Scientific). RNA integrity was assessed using the Agilent 2100 Bioanalyzer (Agilent Technologies). In each sample the RNA integrity number was measured (RINs > 7). For each sample, 100 ng of total RNA were used to prepare cDNA libraries using Illumina RiboZero kits and to construct libraries with NEBNext Ultra directional RNA library prep kit according to the manufacturer's protocols. Samples were sequenced to produce 2 × 75 bp paired-end reads on an Illumina HiSeq. 4000 platform. Adaptor sequences were removed with CUTADAPT version 1.2. and further trimmed with SICKLE version 1.200 (minimum window quality score of 20). This resulted in a mean library size of 18 million (range = 5–50 million) paired-end reads.

High-quality reads were mapped against a draft genome for *M. agrestis* (GenBank Accession no. LIQJ00000000) using TOPHAT version 2.1.0^13^, and a set of predicted gene models was generated using BRAKER2^14^. Mapped reads were counted using FEATURECOUNTS^15^. Further analysis was performed on counts of mapped reads for each gene in R version 3.4.2^16^. These count data were initially filtered to remove unexpressed genes (those genes with fewer than three counts per million across all samples; *n* = 13). Following filtering, library sizes were recalculated and data were normalised to represent counts per million (cpm). No correction for gene length was necessary as all analyses were based on comparisons across (rather than within) samples. Transcript abundance for a particular gene here represents a single, functional measure of its activity across some, mixed, cell population.

In order to validate these RNASeq data, two-step reverse transcription quantitative PCR (Q-PCR) was performed on the same (unstimulated) samples and the expression of four example genes was measured: *Arg1*, *Foxp3, Cd8a* and *Il1b. Foxp3* and *Arg1* primer sequences were designed *de novo* and validated in house. *Arg1* primer sequences were as follows GCAATTGGAAGCATCTCTGG (forward primer sequence) and TGATATCGGTGTGAGGATCC (reverse primer sequence) and *Foxp3* primer sequences were CAGCAGCTGGAGCTGGAAAA (forward primer sequence) and GGACCAGGCTGGGAGAACAC (reverse primer sequence). *Cd8a* and *Il1b* primers were designed *de novo* and supplied by Primer Design (Chandler’s Ford, UK; see^10^ for detailed Q-PCR methodology). Expression levels estimated by RNASeq and Q-PCR were found to be positively correlated for three out of the four example genes (*Arg1*: rho = 0.61, *p* < 0.0001; *Cd8a*: rho = 0.52, *p* < 0.0001; *Il1b*: rho = 0.63, *p* < 0.0001; Supplementary Fig. S1a–c).

In order to maximise the power of our analysis to identify biologically relevant patterns, we focussed on those genes which were expressed at an ‘informative’ level in the spleen prior to and/or following stimulation. Genes expressed at a mean level greater than 200 cpm were considered ‘informative’ (*n* = 1350 or 6%). Of these, 1159 were annotated (see Supplemetary Table S1 for a full list of these genes). A relationship between gene-wise mean expression level and variance is typically found in RNASeq data, with low mean transcript abundance being strongly associated with high variability^17^ and therefore low repeatability (e.g. *Foxp3*; Supplementary Fig. S1d). We chose a threshold of 200 cpm here, because this is the region in which the mean-variance relationship asymptotes for our data i.e. a gene’s variance becomes independent of its mean expression level (Supplementary Fig. S2). This threshold is of a similar magnitude to that reported for a similar dataset, composed of unrelated human individuals, with high levels of biological variation^17^. As weakly expressed genes were removed (minimising heteroscedasticity), log-transformation of data was unnecessary. Both baseline and stimulated samples varied in their expression of informative genes (Supplementary Fig. S3).

### Statistical methods

Genes for which a response to stimulation was observed across individuals were identified, and, as elaborated in the Results, categorised on the basis of (i) the dependence of an individual’s response on its baseline abundance, and (ii) the degree of variability in response across individuals.

#### Baseline-dependence of response

The dependence of an individual’s response on its baseline abundance was quantified by testing the relationship between that individual’s baseline abundance (cpm_base_) and its stimulated abundance (cpm_stim_) using a linear regression (also known as an ordinary least squares or OLS regression), taking the form

$${{\rm{cpm}}}_{{\rm{stim}}} \sim {{\rm{cpm}}}_{{\rm{base}}}$$

as well as a quadratic regression, taking the form

$${{\rm{cpm}}}_{{\rm{stim}}} \sim {{\rm{cpm}}}_{{\rm{base}}}+{{{\rm{cpm}}}_{{\rm{base}}}}^{2}$$

Thus evaluating if stimulated abundance showed a curvilinear relationship with differences in baseline abundance. For approximately one third of genes (*n* = 466), the residuals from both of these regressions deviated significantly from the assumptions of normality and/or homoscedasticity, and a non-parametric Kendall–Theil linear regression was fitted instead.

Regression fits varied from gene to gene (R^2^ ranging from <0.001 to 0.85). As well as evaluating the significance of the quadratic term, the slopes of linear regression fits were tested for whether they were (i) significantly different from zero, and (ii) significantly different from one (we report whether slopes were greater or less than one in the text). Intercepts of linear regression fits were also tested for whether they were significantly different from zero. All resulting *p*-values, used to categorise genes, were adjusted for multiple testing using the Benjamini-Hochberg method^18^.

OLS regression assumes that the variable on the *x*-axis is measured without measurement error. Although this is not true for baseline abundance, by excluding those genes with low mean abundance, and focussing instead on high abundance genes, we were able to minimise this measurement error. As a result, OLS regression was still deemed appropriate, despite a small bias, especially as our goal was not precise slope estimation, but rather, assigning slopes to broad categories, and statistically testing whether slopes were different from reference values^19^.

#### Individual variability in response

Individual variability in response was quantified by comparing the coefficient of variation (CV) for baseline abundances across individuals (CV_base_), and the CV for stimulated abundances across individuals (CV_stim_). Given that an individual’s response is defined as the difference between its baseline and stimulated abundance, a significant difference in the individual variability present at these two levels, either

$${{\rm{CV}}}_{{\rm{base}}} > {{\rm{CV}}}_{{\rm{stim}}}$$

or

$${{\rm{CV}}}_{{\rm{stim}}} > {{\rm{CV}}}_{{\rm{base}}}$$

is indicative of high individual variability in response. As we restricted our analysis to informative genes only, excluding those genes with low mean abundance, it was not necessary to account for the gene-wise mean-variance relationship (see above). Asymptotic tests for the equality of CVs were run using the R package cvequality^20^. All resulting *p*-values, used to further categorise genes, were adjusted for multiple testing using the Benjamini-Hochberg method^18^.

#### Functional annotation

Functional enrichment analysis was performed on gene sets, to uncover their biological functions, using The Database for Annotation, Visualization and Integrated Discovery (DAVID)^21,22^ version 6.8. Only genes which were annotated in the draft genome for *M. agrestis* could be included in gene sets and analysed in this way. The population background against which gene sets were analysed was composed of all annotated genes in the draft genome (*n* = 13426 *M. agrestis* genes mapped to *n* = 14161 *Mus musculus* genes). Benjamini-Hochberg corrected *p*-values and gene counts are reported alongside ontology terms of interest, including Kyoto Encyclopedia of Genes and Genomes (KEGG) pathways^23–25^, to indicate their level of enrichment (significance cut-off = 0.1). A small number of genes (e.g. predicted genes) were unmapped in DAVID and were excluded from the enrichment analysis.

#### Age-specific analysis

In order to begin to investigate the relative importance of genetic variation versus prior stimulation for shaping patterns of variation in response, the same analysis was performed separately on immature and mature voles. As we had more samples from mature voles (*n* = 43) than immature voles (*n* = 19), we randomly sampled the mature population (with replacement) 1000 times and averaged across these samples. The number (immatures) or mean number (matures) of genes in a response category for each of these age classes is presented in the text.

## Results

### Stimulation with an immune agonist causes a widespread response

1150 genes (5% of all genes in the field vole genome and 85% of informative genes, those genes which were more strongly expressed in the spleen) fell into one or more of the response categories set out in Fig. 1. As expected, given that anti-CD3 and anti-CD28 antibodies are known to stimulate T-cell proliferation^12^, they were enriched with genes (hereafter ‘markers’) associated with the T-cell receptor (TCR) signalling pathway (*n* = 26; *p* < 0.001) and other T cell-related terms: positive regulation of T-cell proliferation (*n* = 12; *p* = 0.08), TCR complex (*n* = 7; *p* < 0.01), positive thymic T-cell selection (*n* = 7; *p* = 0.01), negative thymic T-cell selection (*n* = 6; *p* = 0.05) and alpha-beta TCR complex (*n* = 5; *p* < 0.01). For the majority of these markers, a significant positive linear relationship was found between baseline and stimulated abundance (*n* = 844). Only a single marker, *Fam193b*, demonstrated a significant negative linear relationship between baseline and stimulated abundance.

### There are three main categories of response to stimulation

Three main categories of response to stimulation were identified based on the dependence of an individual’s response on its baseline abundance. Each of these categories demonstrates a unique pattern (Fig. 1):

#### Uncorrelated response

Markers for which individuals taken from the wild differ in their baseline abundance, but the responses of different individuals are variable and independent of their baseline, such that the slope of the relationship between baseline and stimulated abundance is not significantly different from zero.

#### Constant response

Markers for which individuals taken from the wild also differ in their baseline abundance, but the responses of different individuals are (on average) constant and independent of their baseline, such that the slope of the relationship between baseline and stimulated abundance is not significantly different from one and the intercept (indicating the level of response) is significantly greater than zero.

#### Baseline-dependent response

Markers for which individuals taken from the wild again differ in their baseline abundance, but the responses of different individuals vary as a function of their baseline, either as a linear function of their baseline (slope significantly different from one), or as a quadratic function of their baseline, where stimulated abundance either increases exponentially as a function of baseline abundance or becomes saturated at some upper limit.

We also identified markers, across these three categories, which displayed high individual variability in response. These highly variable markers can be further divided into two categories (Fig. 1):

#### Convergent response

Markers for which individual variability in baseline abundance is significantly greater than individual variability in stimulated abundance.

#### Divergent response

Markers for which individual variability in stimulated abundance is significantly greater than individual variability in baseline abundance.

### The baseline-dependent response category is most common and is significantly enriched in components of conventional immune pathways

**Figure 2:**
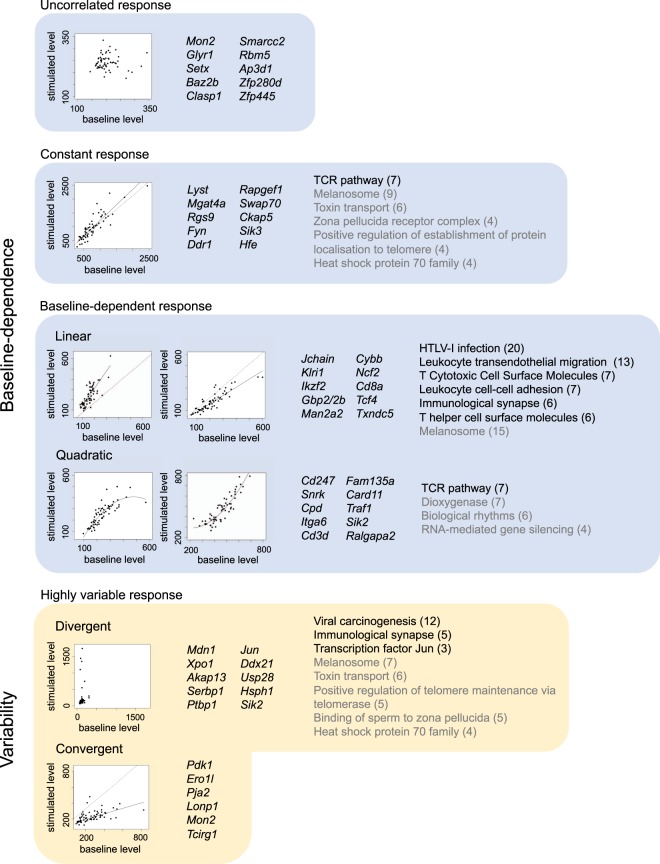
Top 10 markers and enriched ontology terms of interest in each response category. Each box represents a category of response (as in Fig. 1). For each category, top 10 annotated markers for which we had the most confidence in their categorisation (markers were ranked on R^2^ and *p*-values; see Supplementary Tables 2–6 for full set of parameters used to categorise and/or rank these markers) are listed, one or two of these are represented in plots showing stimulated versus baseline abundances (in counts per million) across individuals (solid line indicates significant relationship between baseline and stimulated abundance; dashed line indicates a slope equal to one and the same intercept for reference; note the differing axes of these plots). In the case of the convergent response category, which only included a total of six annotated markers, all markers are listed. Ontology terms of interest, from a functional enrichment analysis performed on all markers within a category (where possible), are also included (immune-related terms in black; non-immune-related terms in grey).

The baseline-dependent response category was the most common (Table 1), and included a majority of markers for which stimulated abundance was a linear function of baseline abundance (*n* = 539), and a remainder for which it was a quadratic function (*n* = 160). The majority of quadratic response markers showed evidence for saturation (*n* = 138), indicating some upper limit on stimulated abundance. Functional enrichment analysis revealed significant enrichment for a number of immune ontology terms in the linear response category, including T helper cell surface molecules (*n* = 6; *p* = 0.08), leukocyte transendothelial migration (*n* = 13; *p* = 0.03) and HTLV-1 infection (*n* = 20; *p* = 0.05). Only the TCR signaling pathway was enriched in the quadratic response category (*n* = 7; *p* = 0.03; Fig. 2).

**Table 1:** Table summarising the number of markers found in each of the three main categories of response to a standardised immune challenge. For each of these categories, the number of convergent and divergent markers is shown.

Response category	Total no. markers	No. convergent	No. divergent
Uncorrelated	47	2	1
Constant	306	0	91
Baseline-dependent	699	5	47

### The uncorrelated response category is least common and lacks enrichment in components of conventional immune pathways

A number of markers showed no evidence for a relationship between baseline and stimulated abundance (*n* = 47; Table 1). For the majority of these, mean abundance was significantly greater for stimulated than for baseline samples (*n* = 39; *p* < 0.05), suggesting that these markers were (on average) responding to stimulation, but to an individually variable degree, independent of baseline abundance. These markers lacked any enrichment for immune-related terms (Fig. 2).

### **A number of markers, including*Zap70*, show particularly high individual variability in response**

**Figure 3:**
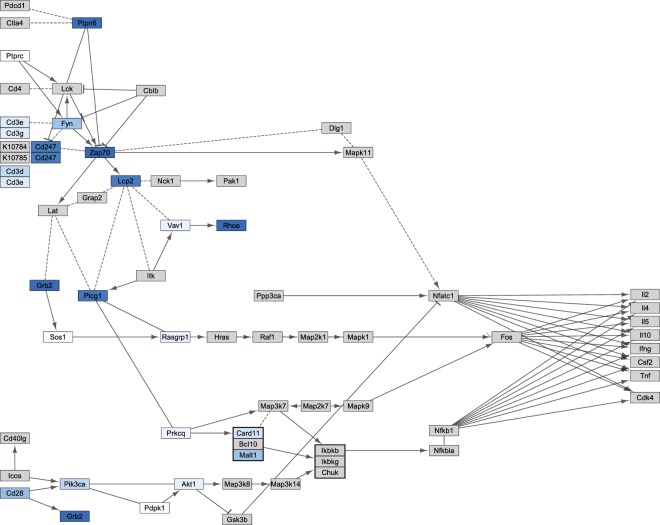
Map of the T-cell receptor signalling KEGG pathway for *Mus musculus*, with the colour of nodes representing the level of individual variability in response to stimulation with anti-CD3 and anti-CD28 antibodies in *Microtus agrestis*. Namely the *p*-value from an asymptotic test for the equality of variance in gene expression levels for baseline and stimulated samples (range = <0.001–0.97). Dark blue indicates high individual variability in response, whereas light blue or white indicates low individual variability in response. Grey nodes represent genes for which no information is available, either because they are unannotated in the *M. agrestis* genome, or because they are weakly expressed in the spleen.

For a number of markers, variability in baseline and stimulated abundance was significantly different, leading to high individual variability in response (*n* = 244). The vast majority of these markers showed a divergent (*n* = 237), rather than a convergent (*n* = 7) response. Within the (stimulated) TCR signalling pathway, the highest level of variability in individual response, and the highest level of divergence, was demonstrated by *Zap70* (Fig. 3). All convergent markers fell into one of the three main categories of response. However, over a third of divergent markers (*n* = 98), did not fall into any of these categories (Table 1), appearing instead as markers which (on average) did not respond to stimulation. There was also no significant difference in the mean abundance of these markers in stimulated and baseline samples (*p* > 0.05).

### Immature voles show more individual variability in response than mature voles

An age-specific analysis showed that high individual variability in response to stimulation (whether divergent or convergent) was more common among immature voles (numbers of markers; divergent = 108; convergent = 6) than mature voles (mean numbers of markers and empirical 95% intervals; divergent = 50 [0–338.2]; convergent = 0.11 [0–1]).

### Response to stimulation is not limited to components of conventional immune pathways

Functional enrichment analysis revealed that non-immune related ontology terms were also enriched in the response categories, including: toxin transport (*n* = 6; *p* = 0.02 in divergent response category; *n* = 6; *p* = 0.05 in constant response category) and dioxygenase (*n* = 7; *p* = 0.002 in baseline-dependent response category). The top convergent response marker, *Pdk1*, is also a component of the insulin signalling pathway (Fig. 2).

## Discussion

The need to better understand variation in immune response in natural populations is now widely accepted^26–29^. Our understanding of immune responses in laboratory settings comes from animals that vary little either genetically or in prior experience. By contrast, animals in natural populations vary (perhaps extensively) in both of these. In this study, we describe the patterns of natural variation in response to a standardised immune challenge in a wild population of rodents. We identify three main categories of response: uncorrelated response, constant response and baseline-dependent response. We also identify markers, across these categories, which show particularly high individual variability in response.

The baseline-dependent response category is the largest. Markers in this category show a relationship between baseline and stimulated abundance across individuals, and their response to stimulation is (to a lesser or a greater extent) dependent on their baseline abundance. In some cases, individuals already expressing the greatest abundance of a marker in their natural setting went on to exhibit the greatest response to stimulation by an agonist. In others, the opposite was true, and these individuals exhibited the smallest response to stimulation. Similarly, previous work on humans has identified baseline (transcriptional) predictors of influenza vaccination response^30,31^. These differences in baseline abundance could be driven by either genetic variation or individual differences in past experience. In humans, genetic determinants of baseline immune cell population frequencies have been identified^32^. Even though the stimulation we describe here was not antigen specific, previous challenge by a pathogen might also lead to changes in the baseline T-cell population within an individual’s spleen, affecting its response to any subsequent challenge. In fact, we find that voles infected with *Babesia microti* (a blood parasite, common in our population^33^) have larger spleens than uninfected voles^34^. This prior experience may prime an individual, enabling a greater response to subsequent challenge (e.g. slope greater than one; Fig. 1). However, individuals may also have an upper limit on the number of immune cells they have available^35,36^. An individual that is already mounting an immune response to a pathogen, and has a large number of activated T cells, could therefore respond less to a similar challenge than an ‘immunologically naïve’ individual (slope less than one; Fig. 1). Membership of the baseline-dependent response category recapitulates the known biology of the immune response (being highly enriched for immune ontology terms). In doing so, it validates the approach we use here, as a way of identifying markers of immune significance.

For both the uncorrelated response category and the constant response categories, individuals varied in their natural abundance of a marker but their response was unrelated to this. For the uncorrelated response category, the majority of markers occurred at a significantly higher mean abundance in stimulated samples than in baseline samples suggesting that they did nevertheless respond to stimulation. This category, which contains a moderate number of markers, also lacks any enrichment for immune-related ontology terms. This suggests that markers in this category are not conventional immune markers but could be of immune significance. We warn against omitting such markers from studies of immune response in the laboratory. They could play an important part in our understanding of the immune response, indicating for example, genetic variation in response among individuals, which is independent of baseline abundance.

The constant response category was the second largest and was moderately enriched for immune ontology terms. Markers in this category showed (on average) a constant response to stimulation across individuals. This pattern may be characteristic of markers that are of critical importance in mounting a successful immune response to a pathogen *in vivo*, and are therefore under tight regulation. For example, one of the top markers in the constant response category*, Fyn*, plays a central role in initiating T-cell activation and expansion^37,38^. *Fyn*-deficient mice have a severely compromised response to staphylococcal enterotoxin B, demonstrating the importance of this gene for mounting a successful response^39^. However, this is not the case for all markers in this category. Some markers showed individual variation around the average (constant) response and, for almost a third, responses of different individuals were highly variable (see below).

Cutting across this categorisation, a large number of markers displayed a pattern in which variation between individuals was particularly strong. We describe two types of such markers, both of which could be used in future studies as indicators of natural variability in immune response. Markers in the less common, convergent, response category showed much greater variation naturally than following stimulation. This pattern may be characteristic of markers showing variable levels of prior activation and some maximum or optimum abundance, resulting in a stabilisation of marker levels across the population following stimulation. We found that convergent patterns were more common among immature voles.

Assuming that individuals converge immunologically over time as they become colonised by the most common pathogens in the environment, we would expect young voles to be more variable in their levels of prior activation, as a result of their shorter and more variable exposure histories^40^. This could make them more likely to converge. Equally though, this could suggest that they are more constrained in the energy they have available (as a result of the competing energetic demands of growth and development) or the number of immune cells they have available (as a result of a developing immune system). Either resource constraint could result in a maximum abundance, also making them more inclined to converge. Due to the costly nature of the immune response, individuals often trade-off their investment in different arms of the immune system^41,42^. Different types of immune response are therefore likely to be associated with different optimum abundances (or regions) and an individual already mounting an immune response, but to a different type of challenge (associated with different cell types), may respond by down-regulating expression.

Divergent markers, which were more common, showed much greater variation following stimulation than there was naturally. This pattern may be characteristic of (but not limited to) markers showing genetic variation in response to the agonist, independent of baseline abundance e.g. subsets of animals that appear similar but respond more strongly to stimulation than others. Our own recent work, where we found an association between polymorphism in a single gene and a marker of a more tolerant immune response^43^, is an example of such genetic variation in response. Further supporting this hypothesis, here, we found more divergent markers among immature voles than mature voles. Assuming that individuals diverge, rather than converge, immunologically over time as a result of the cumulative influence of environmental exposure^4^, we would expect genetic effects to be more easily detectable in younger voles, with shorter exposure histories. Equally, though, divergent patterns could be the result of differences in early life experiences. One would also expect these to be more easily detectable in immature voles.

The divergent category (predominantly) included markers for which individuals made (on average) the same response to stimulation, and markers that did not respond (on average) to stimulation. Standard differential expression analysis would miss the individual variation present in the former group, and would fail to pick up the latter group of markers altogether. Both warn against looking at average (population-level) response, and point instead, to the value of looking at individual-level differences in response. This is particularly important because divergent markers may act as critical regulators of pathways. For example, *Zap70*, which demonstrates particularly high levels of individual variability in response and is centrally located in the TCR signalling pathway, interacts with many other markers (Fig. 3). We suggest that *Zap70* expression could be used as a marker of response in larger studies. Indeed, it is already linked to major seasonal immune variation in wild fish^44^ and is being used as a prognostic marker for B-cell chronic lymphocytic leukemia in humans, with potential implications for determining a patient’s treatment path (recently reviewed in^45^). This example supports our assertion, in this study, that markers for which response is most variable among individuals are likely to be most useful for understanding why individuals vary in susceptibility. Other potential prognostic (or diagnostic) markers, which may have been missed using standard differential expression analysis, may be present in this category and warrant further investigation. Individual markers do not work in isolation though, and the immune system is a ‘multi-faceted defence system’^46^. For example, one study showed that 24 immunological parameters explained 60% of the variation in resistance to streptococcus group B in laboratory mice when combined in a multivariate analysis, providing insight which would have been missed by analysing each immunological parameter separately^47^. Similarly, groups of markers of prognostic and/or diagnostic value may be identified within this category by applying multivariate statistical methods.

The response categories we describe here are based on splenocytes (i) stimulated with anti-CD3 and anti-CD28 antibodies, (ii) sampled at 24 hours and (iii) assayed by RNASeq. Firstly, we chose to use a broad spectrum stimulus, as opposed to a pathogen-specific challenge, as we were looking for broadly applicable response categories. One caveat on our results is that our choice of agonist specifically activates and expands T cells. Our results may therefore exclude or underrepresent host responses that are independent of, or less strongly driven by, T cells. Furthermore, unlike an immune agonist, a pathogen is a dynamic, living organism which can multiply, evolve and interact with the host’s immune response by e.g. evasion or manipulation. In order to fully understand why individuals vary in their susceptibility to infection then, we must consider the pathogen as well as the host’s response^46^. We hope that future work will verify the categories we set out here using other immune agonists or, ideally, a range of pathogen-specific challenges.

Secondly, we sampled our spleen cells at 24 hours post stimulation, but the choice of time point may affect the relative frequency of the response categories reported here. Markers are known to follow different response trajectories, with some immediately responding and reaching peak activation, and others taking longer to reach this point^48^. Sampling at a later time point, then, when the ‘slower’ markers have reached peak activation, may lead to more convergence than reported here. In order to fully account for this temporal variation, multiple time points need to be averaged across. We argue that both time-specific and averaged responses are of functional significance, but hope others will extend our work.

Thirdly, we chose to assay expression by RNASeq, again, in order to give a broad view of the response to stimulation. Previous work has shown that transcript levels generally correlate with protein levels across genes^49^. However, more work is needed to confirm these response categories at this functional level^50^. In future, quantitative PCR (Q-PCR) or protein-level data could be used in order to include weakly expressed markers, which were excluded here as a result of the heteroscedasticity inherent in RNASeq data. Single-cell RNASeq could also be used to quantify differences in individual response resulting solely from differences in cell-specific activity.

Markers that responded to stimulation were not limited to immune pathways as conventionally defined. They included markers involved in energy generation, including the insulin signalling pathway. This is in line with previous studies, which suggest that insulin plays a key role in coordinating an organism’s response to infection, influencing, in particular, the allocation of resources^51,52^. The marker in question, *Pdk1*, was among the top convergent markers. This could be representative of the high levels of variability in the (baseline) nutritional status of individuals in a natural population, coupled with an upper limit on the processes involved in glucose metabolism.

The response categories we presented here, therefore, highlight markers not traditionally associated with immune functions, and offer a promising avenue for identifying potential prognostic (or diagnostic) factors for disease, like *Zap70*. They also point to both genetics and prior experience as important drivers of immune variability, providing the next challenge for understanding why individuals vary in their response to infection. The field population we study has been the subject of extensive previous study on population ecology and pathogen dynamics^33,34,53,54^. Our future work will, therefore, place the patterns of natural variation described here into this context, in order to decompose them into those driven by genetics and prior experience.

## Supplementary information

Supplementary Information.

## Data Availability

RNASeq data are available from the European Nucleotide Archive (study accession number PRJEB23617).
