# Supplementary material for: Transcriptome-wide analysis reveals different categories of response to a standardised immune challenge in a wild rodent
Source: Sci Rep. 2020 May 4;10:7444. doi: 10.1038/s41598-020-64307-7 (PMC7198573; doi:10.1038/s41598-020-64307-7)
Supplement: Supplementary file 1 — Supplementary Information. [file 41598_2020_64307_MOESM1_ESM.pdf]

Supplementary Information for:

Transcriptome-wide analysis reveals different categories of response to a standardised immune challenge in a wild rodent

Klara M. Wanelik, Mike Begon, Elena Arriero, Janette E. Bradley, Ida M. Friberg, Joseph A. Jackson, Christopher H. Taylor, Steve Paterson

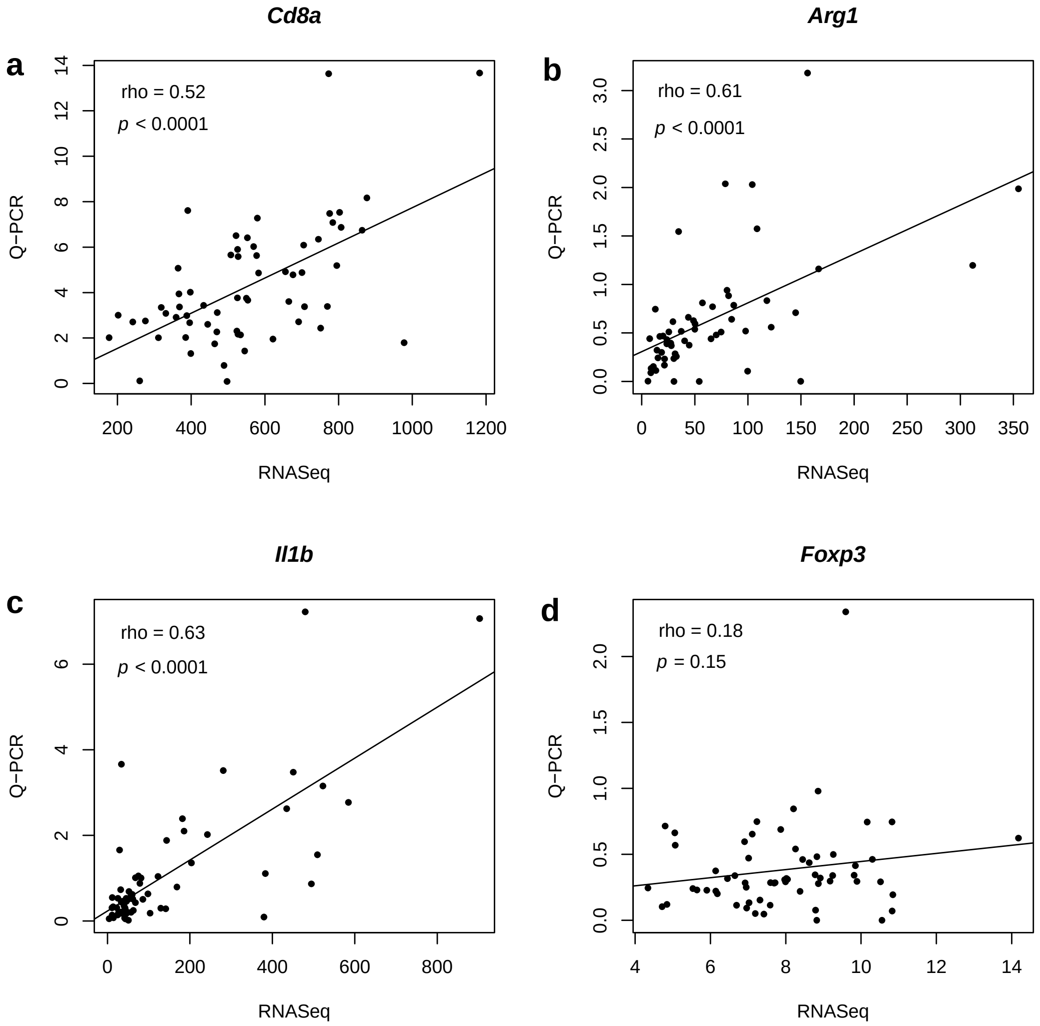

Figure S1. Comparison of expression levels for four genes (a) *Cd8a*, (b) *Arg1*, (c) *Il1b* and (d) *Foxp3* estimated by RNASeq and two-step reverse transcription quantitative PCR (Q-PCR) performed on the same control (baseline) samples in order to validate the RNASeq data. Counts per million (cpm) are plotted on the *x*-axes (RNASeq), and expression levels relative to the house-keeping gene, *Ywhaz*, are plotted on the *y*-axes (Q-PCR). See[1] for detailed methodology. A line of best fit is shown, along with Spearman rank correlation coefficient (rho) and associated significance value. All correlation coefficients are positive, but they vary in their magnitude and

significance. *Cd8a*, *Arg1* and *Il1b* all have significant correlation coefficients of high magnitude, indicating highly repeatable expression levels. However, *Foxp3* has a non-significant correlation coefficient of low magnitude, indicating less repeatable expression levels. This is because it is expressed at a much lower level than the other three genes. In our analysis, we removed weakly expressed genes in order to ensure repeatability (see main text for further details).

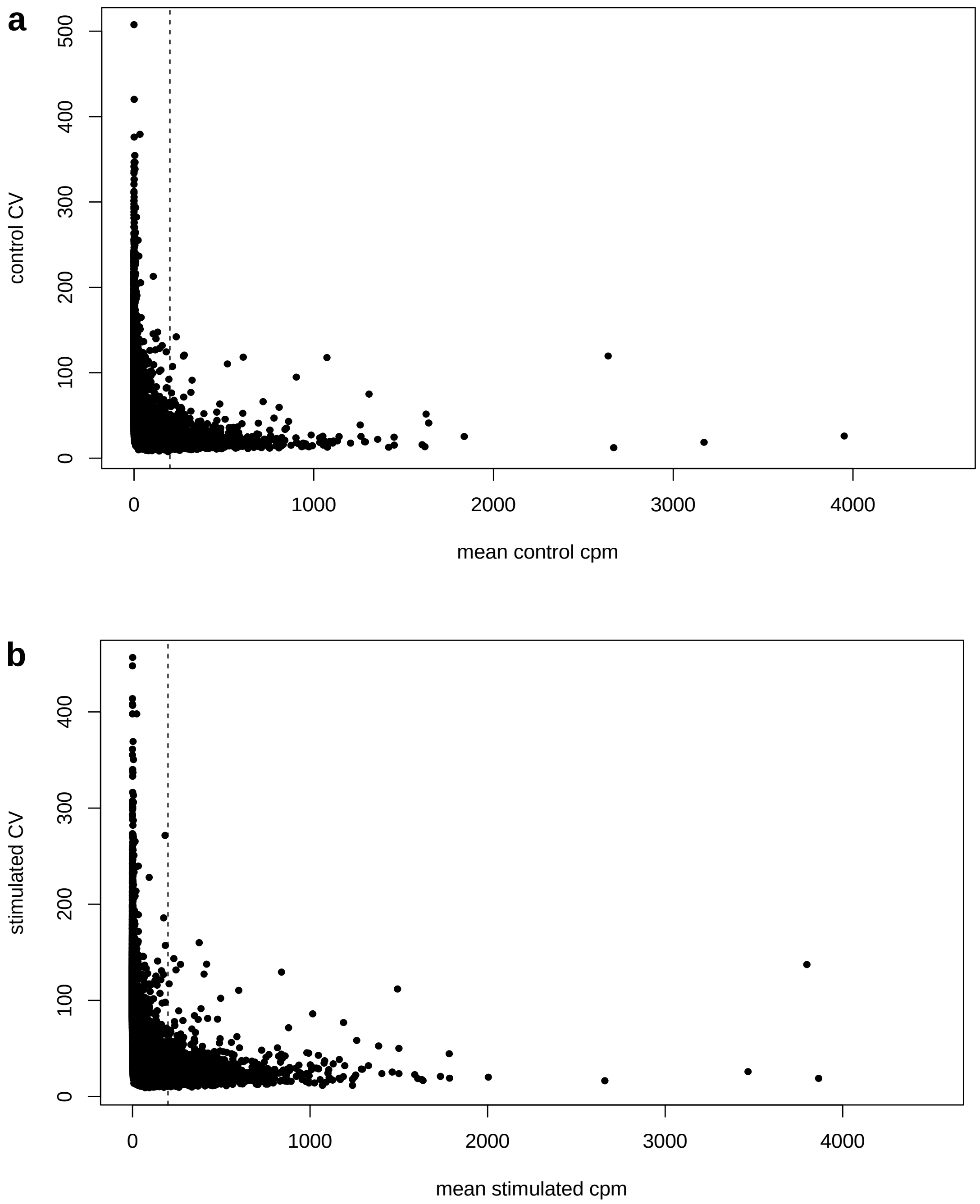

Figure S2. Relationship between gene-wise means and coefficients of variation (CV) for expression levels in (a) control (baseline) samples and (b) stimulated samples. Weakly expressed genes were omitted from the analysis. A threshold of 200 counts per million (cpm) in baseline samples and/or stimulated samples (indicated by dashed line) was chosen because this is the region in which the mean-variance relationship asymptotes i.e. a gene's variance becomes independent of its mean expression level, and other variables used to categorise genes also become independent of mean expression levels (as tested by Spearman's rank correlation tests).

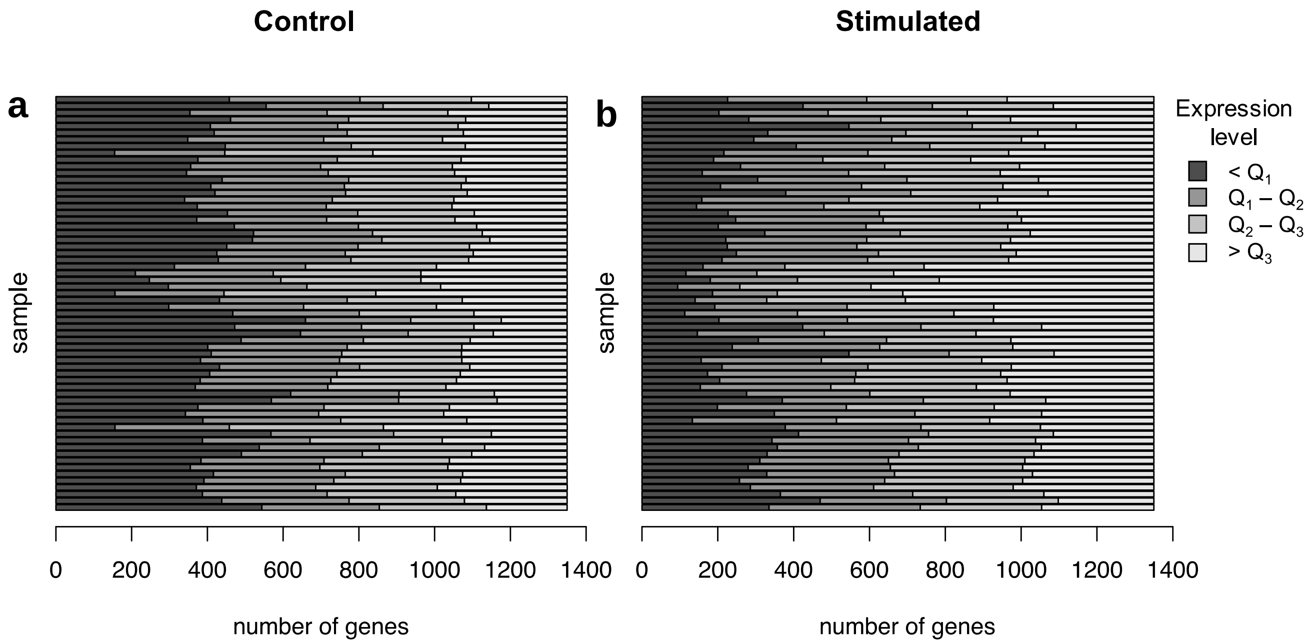

Figure S3. Variation in gene expression between (a) control (baseline) samples, and (b) stimulated samples. Each horizontal bar represents a single sample, and is divided into sections to indicate the number of informative genes (mean expression level > 200 cpm; *n* = 1350) expressed at four different levels (these four levels are defined by the quartiles of our gene expression dataset: ■ : < Q_1; ■ : $Q_1 – Q_2$; ■ : $Q_2 – Q_3$; □ : > Q_3).

Table S1. List of (annotated) genes included in our analysis. These were genes expressed at an informative level (mean expression level > 200 cpm) in spleen prior to and/or following stimulation with an immune agonist. In order to identify these genes, Illumina reads were mapped against a draft genome for *Microtus agrestis*. Where reads mapped onto multiple genes, or reads mapped onto a gene with multiple possible annotations, these are shown on a single line.

Gene symbol			
Hnrnph1 Hnrnph2	*Nup153*	*Lcp1*	*Ctsz*
Edem3	*Bc005537*	*Crebrf*	*Gnas*
Ints8	*Fam65b*	*Snd1*	*Gmip*
Trp53inp1	*Lpin1*	*Slc38a10*	*Mau2*
Ppp1r15b	*Flna*	*Zfp445*	*Upf1*
Tnfaip3	*Gdi1*	*Fyco1*	*Kmt2d*
Heca	*Ccnt1*	*P4hb*	*Lmbr1l*
Marf1	*Glyr1*	*Mafg*	*Mbtps1*
Trim24	*Ubn1*	*Fasn*	*Leng8*
Zc3hav1	*Crebbp*	*Csnk1d*	*Edem1*
Gigyf1	*Klri1*	*Dhx9*	*Etnk1*
Lrch4 Gm20605	*Actr3*	*Skil*	*Cybb*
Tfrc	*Arhgap17*	*Txnip*	*Susd6*
Ubxn7	*Vps13a*	*Dennd5a*	*Smg1*
Wnk1	*Poldip3*	*Jmjd1c*	*Cflar*
Myo1g	*Tcf20*	*Itm2b*	*Clk1*
Il16	*Lrrc8c*	*Osbpl8*	*Zfp292*
Cxcr4	*Clk3*	*Fbxo38*	*Sf3b1*
Fgd3	*Gpbp1*	*Ghitm*	*Chd6*
Fam120a	*Stk17b*	*Pdk1*	*Plcg1*
Prr14l	*Rpl18*	*Itga6*	*Scarb2*
Gpbp1l1	*Syk*	*Rnf149*	*Ccni*
Samhd1	*Sp3*	*Cul3*	*Anapc5*
Rpn2	*Akap11*	*Tnfrsf1b*	*Clock*
Adam17	*Evl*	*Vps13d*	*Rapgef1*
Arpc1a Arpc1b	*Yy1*	*Vcp*	*Ahcyl1*
Trrap	*Wars*	*Fas*	*Strip1*
Stat1	*Ikbkap*	*Atp5a1*	*Usp22*
Stat1	*Rpl12 Gm16519*	*Mark3*	*Fam135a*
Gls	*Dlst*	*Rcor1*	*Il17ra*
Arf6	*Arel1*	*Akt1*	*Kdm5a*
Slc44a2	*Nampt*	*Sbf1*	*Xpot*
Jak1	*Cerk*	*Pnisr*	*Birc6*
Icam1	*Map3k1*	*Api5*	*Pacs1*
Smg6	*Skiv2l2*	*Rpl14*	*Grk2*
Tgfbr2	*Il6st*	*Fndc3a*	*Kdm2a*
Cct5	*Slc20a1*	*Csnk2a1 Gm10031*	*Gltscr2*
March6	*Arrdc4*	*Dmxl1*	*Trim33*
Pkn1	*Vav3*	*Atf4*	*Grcc10*

Ddx39 Ddx39b	*Gpi1*	*Sun2*	*Ptpn6*
Adgre5	*Uba2*	*Tnrc6b*	*Chd4*
Dennd2d	*Rpl37a*	*Ppm1a*	*Arpc2*
Safb2	*Tmem33*	*Msn*	*Gbp2 Gbp2b*
Nckap1l	*Zcchc6*	*Ogdh*	*Lbr*
Ncor1	*Cnot2*	*Dennd4a*	*Cd6*
Plekho2	*Clasp1*	*Prkch*	*Cltc*
Fryl	*Ahctf1*	*Hif1a*	*Med13*
Gatad2b	*Adcy7*	*Fam91a1*	*Sh3bgrl*
Pbxip1	*Zfp217*	*Ik*	*Fyb*
S100a9	*Phf3*	*Dazap2*	*Rictor*
S100a8	*Itsn2*	*Hmgcs1*	*Cd82*
Cct7	*Ctsh*	*Fam49b*	*Pja2*
Pik3ap1	*Tbc1d2b*	*Asap1*	*Mapk8ip3*
Tm9sf3	*Irf2bp2*	*Ankrd13a*	*Cramp1l*
Snx3	*Psme4*	*Smad7*	*Unkl*
Pcmtd1	*Bach1*	*Trpc4ap*	*Pdpk1*
St8sia4	*Supt20*	*Wwp2*	*Pkd1*
D16ertd472e	*Zfp740*	*Usp7*	*Rbl2*
Ldlrap1	*Itgb7*	*Setd2*	*Jchain*
Wasf2	*Ddit4*	*Atad2b*	*Rufy3*
Pigv	*Vsir*	*Cd247*	*Slc41a1*
Arid1a	*Psap*	*Ranbp2*	*Psmd2*
Akap13	*Sik3*	*Lims1*	*Eif4g1*
Htt	*Nbr1*	*Zcchc11*	*Srcap Gm42715*
Aebp2	*Gpatch8*	*Eps15*	*Eif3c*
Uba1	*Sptan1*	*Osbpl9*	*Coro6*
Zap70	*Zer1*	*Nrd1*	*Ypel3*
Tmem131	*Hsph1*	*Rpn1*	*Mvp*
Mgat4a	*Eef1b2*	*Cnbp*	*Sept1*
Kansl3	*Nfrkb*	*Dip2b*	*Itgal*
Clint1	*Ptbp3*	*Usp9x*	*Mgea5*
Rnf145	*Ugcg*	*Ddx3x D1pas1*	*Ldb1*
Dicer1	*Ai314180*	*Cit*	*Cbfb*
Anxa6	*Tsc22d3*	*Usp34*	*Nfatc3*
Tnip1	*Plekhb2*	*Nr2c2*	*Ctcf*
Nub1	*Prkacb*	*Cnot1*	*Carmil2*
Hipk1	*Thbs1*	*Pten*	*Edc4*
Otud4	*Slc12a6 Gm21985*	*Tsc1*	*Cnot6l*
Abce1	*Baz1a*	*Ralgds*	*Akirin1*
Gapvd1	*Mllt10*	*Ddx24*	*Rab6a Rab6b*
Ero1l	*Ubqln1*	*Cyp51*	*Baz2b*
Kmt2c	*Gak*	*Gm15800*	*Herc4*
Uba6	*Prkcq*	*Trafd1*	*Arid2*
Mkln1	*Gdi2*	*Nup205*	*Zfp36l2*
Ube2h	*Fam208b*	*Snrk*	*Vhl*

Wipf1	*Prrc2b*	*Nktr*	*Irak2*
Rif1	*Lcn2*	*Rpl27a*	*Adipor1*
Sorl1	*Ywhaz*	*Arhgef1*	*Kdm5b*
Cdk13	*Azin1*	*Bmp2k*	*Zc3h11a*
Zcchc7	*Rnf19a*	*Chd2*	*Gm38394 Zbed6*
Atp6v1b2	*Vps13b*	*Ppp6r1*	*Btg2*
Pbrm1	*Ubr5*	*Oaz1*	*Fam76b*
Setdb2	*Rabgap1l*	*Dot1l*	*Setx*
Rlf	*Mkrn1*	*Ap3d1*	*Ighm*
4932438a13rik	*Klhl11*	*Btbd2*	*Ctsb*
Rbm33	*Cul1*	*Csnk1g2*	*Sesn3*
Akap13	*Ccnt2*	*Stk11*	*Cd74*
Sept9	*Atm*	*Sbno2*	*Csnk1a1*
Tnrc6c	*Luc7l3*	*Hmha1*	*Vav1*
Tmc6	*Spag9*	*Abca7*	*Pdcd6ip*
Ptger2	*Cic|cic*	*Cnn2*	*Etf1*
Mtmr1	*Ppp4r1*	*Ptbp1*	*Hspa9*
Cep350	*Pak2*	*S1pr4*	*Stk24*
Tor1aip1	*Usp32*	*Prkcd*	*Gpr183*
Ier5	*Bptf*	*Sptbn1*	*Tm9sf2*
Aftph	*Helz*	*Epb41*	*Pan3*
Foxo1	*Tnfrsf14*	*Atr*	*Tmem123*
Rb1cc1	*Pank4*	*Rasa2*	*Birc3*
Pds5a	*Nadk*	*Rabgap1l*	*Mcm6*
Rhoh	*Ccnl1*	*Fip1l1*	*Fam214a*
Arap1	*Dvl1*	*Atp13a2*	*Mapk6*
Inppl1	*Fam132a*	*Rcc2*	*Clasp2*
Numa1	*Sdf4*	*Arhgap9*	*Ubp1*
Eif4g2	*Slc25a36*	*Lrp1*	*Herc2*
Samsn1	*Pde4b*	*Stat6*	*Hist2h2bb*
Pcnt	*Surf4*	*Zzef1*	*Pnrc1*
Sik2	*Fermt3*	*Nacc1*	*Ube2j1*
Ppp2r1b	*Atg2a*	*Fnbp4*	*Mdn1*
Frmd8	*Pygm| Sf1*	*Rnf13*	*Map3k7*
Ehbp1l1	*B2m*	*Ube3a*	*Hpse*
Pcnx3	*Arfgef2*	*Tnpo2*	*Fnbp1*
Sf3b2	*Ncoa3*	*Junb*	*Mki67*
Peli1	*Znfx1*	*Atg9a*	*Eif4a1 Eif4a2*
Vps54	*Prex1*	*Fam134a*	*Arhgef6*
Lrrc58	*Olfr609*	*Mfn1*	*Ints6l*
Thoc2 Bc005561	*Ccdc186*	*Pik3ca*	*Rel*
Stag2	*Jun*	*Trim25*	*Card11*
Tmpo|tmpo	*Mysm1*	*Pik3cg*	*Hipk3*
Khnyn	*Dock8*	*Hbp1*	*Cd44*
Ssb	*Ccl22*	*Txndc5*	*Fbxo3*
Ubr3	*Nlrc5*	*Sema4d*	*Sash3*

Anxa11	*Amfr*	*Lrba Nbea*	*Atp2b1*
Kpna1	*Hnrnpl*	*Msl1*	*Btg1 Btg1-ps1 Btg1-ps2*
Cpsf6	*Actn4*	*Casc3*	*Supt16*
Lyz1 Lyz2	*Smpdl3a*	*Top2a*	*Chd8*
9530003j23rik	*Cldnd1*	*Tcf12*	*Pde4d*
Ccnl1	*Plin2*	*Zfp280d*	*Btaf1*
Tiparp	*Dennd4c*	*Rasgrp1*	*Rasal3*
Ssr3	*Whsc1*	*Unc13d*	*Akap8*
Ap1g1	*Golph3*	*Ube2o*	*Brd4*
Sf3b3	*Zfr*	*Grb2*	*Traf3ip3*
Aars	*Pum1*	*Tagap Tagap1*	*Brap*
Glg1	*Ptp4a2*	*Sytl3*	*Atp2a2*
Rfwd3	*Laptm5*	*Ezr*	*Klrd1*
Eprs	*Pkm Pklr*	*Rad21*	*Klrk1*
Chd1	*Arih1*	*Eif3h*	*Slmap*
Pum2	*Tet3*	*Vps4b*	*Arf4*
Atp6v1a	*Mob1a*	*Actr1a*	*Arhgef3*
Tgfb1	*Mthfd2*	*Ikzf1*	*Qk*
Hnrnpul1	*Agfg1*	*Ppp6r3*	*Picalm*
Sos1	*Fyn*	*Tcirg1*	*Crebzf*
Senp6	*Golga4*	*Crem*	*Dhx15*
Tcp11l2	*Asxl1*	*Plekha2*	*Otulin*
Ptprc	*Smc6*	*Tacc1*	*Hsp90b1*
Fkbp8	*Dock10*	*Whsc1l1*	*Kpnb1*
Smap2	*Dock10*	*Rbbp6*	*Mllt6*
Itpkb	*Cep250*	*Sec16a*	*Lasp1*
Pnn	*Cpne1*	*Phtf2*	*Rpl19*
Fbxl5	*Lrrfip1*	*Rsbn1l*	*Nfe2l1*
Lipa Lipm	*Mga*	*Ep300*	*Klf6*
Tmbim6	*Zfp106*	*Lars*	*Akna*
Tomm34	*Cul4a*	*Bmt2*	*Trip12*
Ttc7	*Erap1*	*Kmt2e*	*Tln1*
Paxbp1	*Wtap*	*Eif4g3*	*Ythdf3*
Ifnar1	*Tmf1*	*Usp48*	*Zfp871*
Son	*Bod1l*	*Hp1bp3*	*Atp13a3*
Smc4	*Setd5*	*Trp53*	*Baz1b*
Kpna4	*Arpc4*	*Kdm6b*	*Prrc2c*
Tax1bp1	*Saraf*	*Chd3*	*Suco*
Prpf4b	*Eif3a*	*Per1*	*Cd28*
Ctnnb1	*Irf1*	*Rnf222*	*Icos*
Glud1	*Aff4*	*Usp25*	*Ccar1*
Wdfy3	*Hspa4*	*Dido1*	*Cdv3*
Jak1	*Tcf7*	*Matr3*	*Ccng2*
Tapbp	*Cdc42se2*	*Tmem173*	*Prpf8*
B3galt4	*Rapgef6*	*Kat6a*	*Cpd*
Vps52	*Fnip1*	*Sh3kbp1*	*Ssh2*

Brd2	*Srgn*	*Rnf115*	*Rpl23a Rpl23a-ps3*
Tap1	*Ddx21*	*Rc3h2*	*Supt6*
Psmb8	*Itgb1*	*Smad5*	*Inpp5k*
Plxnc1	*Ofd1*	*Gnl3l*	*Crk*|*crk*
Itga4	*Rbm26*	*Zc3h10*	*Evi2b Gm21975*
Ralgapb	*Fbxo11*	*Esyt1*	*D930015e06rik*
Ash1l	*Acvr1c*	*Smarcc2*	*Topbp1*
2810403a07rik	*Klhl6*	*Cs*	*Apc*
Plec	*Inpp4b*	*Arid5b*	*Brd8*
Parp10	*Parp1*	*Mta2*	*A230046k03rik*
Mroh1	*Prkar1a*	*Ganab*	*Tbc1d17*
Selenop	*Gna13*	*Ubxn1*	*Top2b*
Serbp1	*Rgs9*	*Nxf1*	*Xpc*
Tbl1xr1	*Yod1*	*Slc3a2*	*Ptk2b*
Piezo1	*Ranbp9*	*Trim56*	*Ddi2 Rsc1a1 Gcc2*
Sec63	*Hdlbp*	*Ireb2*	*Ogt*|*ogt*
Mtmr3	*Hdac5*	*Parvg*	*Taf1*
Atf7ip	*Slc4a1*	*Add3*	*Med12*
Med13l	*Ist1*	*Sympk*	*Gm20489*
Klf13	*Atxn1l*	*Clptm1*	*Hspa5*
Topors	*Mdm4*	*Stk26*	*Atp5b*
Os9	*Ncf2*	*Mark2*	*Srrm2*
Ctdsp2	*Smg7*	*Nbeal2*	*Nr3c1*
Clic1	*Iws1*	*Tlk2*	*Fbxl20*
Bag6	*Lrp10*	*Strada*	*Cdk12*
Prrc2a	*Haus4*	*Ddx42*	*Ikzf3*
Slc38a2	*Acin1*	*Cd8a*	*Jak3*
Scaf11	*Tspan13*	*Eif2ak1*	*Fcho1*
Rrp1	*M6pr*	*Usp28*	*Mast3*
Sik2	*Slc2a1*	*Esyt2*	*Jund*
Itgb2	*Sars*	*Acsl4*	*Fam193a*
Pttg1ip	*Ddx5*	*Vps16*	*Add1*
Trpm2	*Xpo7*	*Snrnp200*	*Arl5b*
Pfkl	*Max*	*Dusp2*	*Mtdh*
Brd1	*Zbtb25*	*Prdm2*	*Pip5k1a*
Ggnbp2	*Zbtb1*	*Stat3*	*Smek1*
Slc2a3	*Sptb*	*Fam134c*	*Psd4*
Dennd3	*Lpin2*	*Rbm15*	*Ncoa2*
Ikzf2	*Hdac7*	*Sf3a1*	*Tram1*
Ciita	*Swap70*	*Ckap5*	*Tube1*
Hcfc1	*Ipo7*	*Arfgef1*	*Ddr1*
Pik3r1	*Uhmk1*	*Vcpip1*	*Ifrd1*
Dync1h1	*Kmt2b*	*Traf1*	*Dgka*
Tnpo1	*Ip6k1*	*Kif5b*	*Mgat1*
Fcho2	*Rnf123*	*Svil*	*Cct2*
Dock2	*Rhoa*	*Trbc1 Trbc2*	*Sp1*

Lcp2	*Usp4*	*Csnk1g3*	*Naa15*
Riok3	*Usp19*	*Cep192*	*Ralgapa2*
Ubr2	*Qars*	*Hfe*	*Phf8*
Gtpbp2	*Gnai1 Gnai2 Gnai3*	*Tpp2*	*Huwe1*
Xpo4	*Rbm5*	*Snx5*	*Smc1a*
Lbh	*St6gal1*	*Cap1*	*Kdm5c*
Stt3b	*Nfat5*	*Uprt*	*Tnrc6a*
Dnajc5	*Dst*	*Srsf11*	*Ptpn1*
Mycbp2	*Usp9x*	*Arhgap30*	*Myo15*
Sla	*Cct4*	*Usf1*	*Flii*
Phf20l1	*Xpo1*	*Nup107*	*Abca2*
Kras	*Slc4a7*	*Hivep2*	*Ubr4*
Arhgap15	*Kdm4a*	*Utrn*	*Ubr4*
Brox	*Szt2*	*Arhgdib*	*Igf2r*
Aida	*Gnptab*	*Jade2*	*Tcp1*
Gm2a	*Ythdc1*	*Clk4*	*Smchd1*
Papola	*Ctss*	*Ep400*	*Trpm7*
Pik3cd	*Rabggtb*	*Furin*	*Usp8*
Pgd	*Ilf3*	*Man2a2*	*Sell*
Lnpep	*Prkcsh*	*Iqgap1*	*Foxp1*
Cmip	*Smarca4*	*Anapc1*	*Atrx*
Dgkd	*Dnm2*	*Mcm7*	*Dyrk1a*
Inpp5d	*Myo9b*	*Emsy*	*Larp1*
Cep135	*Tet2*	*Rac2*	*Malt1*
Dmtf1	*Rasa1*	*Myh9*	*Ccpg1*
Arglu1	*Rassf5*	*Ddx17*	*Rab27a*
Gprin3	*Irf8*	*Eif3l*	*Cdc25b*
Mical1	*Cotl1*	*Kdm6a Uty*	*Rassf2*
Foxo3	*Sdcbp*	*Fgfr1op2*	*Smox*
Cd164	*Smc3*	*Def6*	*Canx*
Mier1	*Srebf2*	*Ppard*	*Sqstm1*
Rsf1	*Rangap1*	*Rpl10a*	*Usp15*
Ctsd	*Copb1*	*Stk38*	*Mon2*
Lsp1	*Kif13b*	*Pim1*	*Serinc1*
Cd81	*Gcn1l1*	*Itpr3*	*Kdm1a*
Pitpnm2	*Mlec*	*Gclc*	*Srrm1*
Mlxip	*Ctdspl2*	*Gtf3c1*	*C1qc*
Atp6v0a2	*Cct3*	*Xpo6*	*Zmynd11*
Sbno1	*Smg5*	*Hbs1l*	*Arid4a*
Clip1	*Klhl24*	*Ddx23*	*Arhgap25*
Zcchc8	*Lgmn*	*Mbnl1*	*Arap2*
Hip1r	*Rev3l*	*Cds2*	*Usp47*
Zswim8	*Fyn*	*Gpcpd1*	*Ppil2*
Sec24c	*Sin3a*	*Vps13c*	*Pi4ka*
Ccdc50	*Slamf6*	*Usp3*	*Rpl36 Rpl36-ps3 Gm8973*
Tmem2	*Cd48*	*Herc1*	*Syne1*

Ppp1r9b	*Tagln2*	*Ppib*	*Rftn1*
Kat7	*Dcaf8*	*Cdc27*	*Lonp1*
Parp4	*Bcl11b*	*Fubp1 Khsrp*	*Satb1*
Zmym2	*Kdm3a*	*Rab10*	*Phc3*
Entpd4 Gm21685	*0610030e20rik*	*Cd53*	*Mbd2*
Fam46c	*Polr1a*	*Plcl2*	*Tcf4*
Ddx46	*Ptcd3*	*Plek*	*Cdkn1b*
Fam193b	*Abcc1*	*Ric1*	*Stxbp2*
Grk6	*Stk10*	*Jak2*	*Rasa3*
Nsd1	*Nup210*	*Rsbn1*	*Supt5*
Mms19	*Arid4b*	*Ptpn12 Ptpn22*	*Zfc3h1*
Ppm1b	*Lyst*	*Gabarap*	*Plcg2*
Cbl	*Pfdn5*	*Polr2a*	*Zfp638*
Rps25	*Zfp335*	*Stx7*	*Pcm1*
Ddx6	*Ctsa*	*Pde3b*	*Erbin*
Arcn1	*Ankle2*	*Nipbl*	*Sltm*
Kmt2a	*Hba-x*	*Rfx7*	*Ndfip1*
Cd3g	*Luc7l*	*Avl9*	*Diaph1*
Cd3d	*Axin1*	*Pik3c2a*	*Zfp36l1*
Cd3e	*Gm6768 Ncoa4*	*Cand1*	*Usp24*
Pcnx	*Hmgcr*	*Inpp5f*	

Table S2. Table summarising selected parameters for the top 10 annotated markers in the uncorrelated response category. Parameters in bold were used to categorise and/or rank markers within this category.

**Gene symbol**	Coeff for slope	**Adj *p*-value for slope**	R_2
Mon2	-0.02	**0.85**	< 0.001
Glyr1	0.04	**0.80**	< 0.01
Setx	0.06	**0.72**	< 0.01
Baz2b	0.06	**0.71**	< 0.01
Clasp1	-0.06	**0.62**	< 0.01
Smarcc2	0.08	**0.59**	< 0.01
Rbm5	0.10	**0.47**	0.01
Ap3d1	0.10	**0.45**	0.01
Zfp280d	0.10	**0.44**	0.01
Zfp445	0.15	**0.41**	0.01

Table S3. Table summarising selected parameters for the top 10 annotated markers in the constant response category. Parameters in bold were used to categorise and/or rank markers within this category.

**Gene symbol**	Coeff for intercept	**Adj *p*-value for intercept**	Coeff for slope	**Adj *p*-value for slope**	**Adj *p*-value for slope diff from 1**	**R^2**
Lyst	160.2	**0.03**	1.07	**< 0.001**	**0.47**	**0.76**
Mgat4a	50.1	**0.02**	0.93	**< 0.001**	**0.46**	**0.70**
Rgs9	59.7	**0.02**	1.02	**< 0.001**	**0.87**	**0.66**
Fyn	48.2	**0.02**	0.84	**< 0.001**	**0.09**	**0.63**
Ddr1	343.7	**< 0.001**	0.96	**< 0.001**	**0.72**	**0.62**
Rapgef1	71.0	**0.04**	0.92	**< 0.001**	**0.49**	**0.60**
Swap70	91.5	**< 0.01**	0.85	**< 0.001**	**0.21**	**0.55**
Ckap5	62.6	**0.01**	1.03	**< 0.001**	**0.88**	**0.54**
Sik3	105.1	**0.02**	1.05	**< 0.001**	**0.78**	**0.53**
Hfe	73.0	**0.02**	0.79	**< 0.001**	**0.08**	**0.50**

Table S4. Table summarising selected parameters for the top 10 annotated markers in the baseline-dependent response category. Parameters in bold were used to categorise and/or rank markers within this category.

Quadratic

**Gene symbol**	Coeff for quadratic term (× 10^{-3})	**Adj *p*-value for quadratic term**	**R^2**
Cd247	2.3	**0.01**	**0.80**
Snrk	6.2	**0.03**	**0.75**
Cpd	2.3	**0.04**	**0.75**
Itga6	5.0	**< 0.01**	**0.73**
Cd3d	1.5	**0.01**	**0.71**
Fam135a	-3.7	**< 0.001**	**0.70**
Card11	-4.1	**< 0.001**	**0.67**
Traf1	5.2	**< 0.01**	**0.65**
Sik2	-2.3	**0.02**	**0.65**
Ralgapa2	-3.8	**< 0.01**	**0.62**

Linear

**Gene symbol**	Coeff for slope	**Adj *p*-value for slope**	**Adj *p*-value for slope diff from 1**	**R^2**
Jchain	0.86	**< 0.001**	**< 0.01**	**0.86**
Klri1	1.21	**< 0.001**	**< 0.01**	**0.85**
Ikzf2	1.23	**< 0.001**	**< 0.01**	**0.85**
Gbp2/2b	0.70	**< 0.001**	**< 0.001**	**0.78**
Man2a2	1.30	**< 0.001**	**< 0.01**	**0.78**
Cybb	0.75	**< 0.001**	**< 0.001**	**0.74**
Ncf2	0.70	**< 0.001**	**< 0.001**	**0.68**
Cd8a	0.77	**< 0.001**	**< 0.01**	**0.68**
Tcf4	1.39	**< 0.001**	**0.01**	**0.65**
Txndc5	0.71	**< 0.001**	**< 0.001**	**0.65**

Table S5. Table summarising selected parameters for all markers in the convergent response category. Parameters in bold were used to categorise and/or rank markers within this category (CV = coefficient of variation).

**Gene symbol**	CV for baseline abundance	CV for stimulated abundance	**Adj *p*-value for equality of variance**
Pdk1	49.87	29.51	**< 0.01**
Ero1l	61.95	36.24	**< 0.01**
Pja2	26.91	17.21	**0.01**
Lonp1	29.73	19.41	**0.02**
Mon2	18.04	12.45	**0.03**
Tcirg1	36.91	25.47	**0.05**

Table S6. Table summarising selected parameters for the top 10 annotated markers in the divergent response category. Parameters in bold were used to categorise and/or rank markers within this category (CV = coefficient of variation).

**Gene symbol**	CV for baseline abundance	CV for stimulated abundance	**Adj *p*-value for equality of variance**
Mdn1	18.80	91.43	**< 0.001**
Xpo1	14.08	34.81	**< 0.001**
Akap13	15.66	38.25	**< 0.001**
Serbp1	15.28	37.41	**< 0.001**
Ptbp1	11.97	27.40	**< 0.001**
Jun	24.99	65.00	**< 0.001**
Ddx21	17.28	40.57	**< 0.001**
Usp28	14.60	33.30	**< 0.001**
Hsph1	39.11	143.35	**< 0.001**
Sik2	27.99	68.78	**< 0.001**

References

1. Wanelik, K. *et al.* IgE receptor polymorphism predicts divergent, sex-specific inflammatory modes and fitness costs in a wild rodent. Preprint at https://www.biorxiv.org/content/10.1101/841825v1 (2019).
